# Predicting willingness to donate blood based on machine learning: two blood donor recruitments during COVID-19 outbreaks

**DOI:** 10.1038/s41598-022-21215-2

**Published:** 2022-11-10

**Authors:** Hong-yun Wu, Zheng-gang Li, Xin-kai Sun, Wei-min Bai, An-di Wang, Yu-chi Ma, Ren-hua Diao, Eng-yong Fan, Fang Zhao, Yun-qi Liu, Yi-zhou Hong, Ming-hua Guo, Hui Xue, Wen-biao Liang

**Affiliations:** 1 Jiangsu Province Blood Center, Nanjing, Jiangsu People’s Republic of China; 2 Yangzhou Blood Station, Yangzhou, Jiangsu People’s Republic of China; 3 School of Computer Science and Engineering, Southeast University, Nanjing, Jiangsu People’s Republic of China; 4 Nanjing Foreign Language School, Nanjing, Jiangsu People’s Republic of China

**Keywords:** Translational research, Computer science

## Abstract

Machine learning methods are a novel way to predict and rank donors' willingness to donate blood and to achieve precision recruitment, which can improve the recruitment efficiency and meet the challenge of blood shortage. We collected information about experienced blood donors via short message service (SMS) recruitment and developed 7 machine learning-based recruitment models using PyCharm-Python Environment and 13 features which were described as a method for ranking and predicting donors’ intentions to donate blood with a floating number between 0 and 1. Performance of the prediction models was assessed by the Area under the receiver operating characteristic curve (AUC), accuracy, precision, recall, and F1 score in the full dataset, and by the accuracy in the four sub-datasets. The developed models were applied to prospective validations of recruiting experienced blood donors during two COVID-19 pandemics, while the routine method was used as a control. Overall, a total of 95,476 recruitments via SMS and their donation results were enrolled in our modelling study. The strongest predictor features for the donation of experienced donors were blood donation interval, age, and donation frequency. Among the seven baseline models, the eXtreme Gradient Boosting (XGBoost) and Support vector machine models (SVM) achieved the best performance: mean (95%CI) with the highest AUC: 0.809 (0.806–0.811), accuracy: 0.815 (0.812–0.818), precision: 0.840 (0.835–0.845), and F1 score of XGBoost: 0.843 (0.840–0.845) and recall of SVM: 0.991 (0.988–0.994). The hit rate of the XGBoost model alone and the combined XGBoost and SVM models were 1.25 and 1.80 times higher than that of the conventional method as a control in 2 recruitments respectively, and the hit rate of the high willingness to donate group was 1.96 times higher than that of the low willingness to donate group. Our results suggested that the machine learning models could predict and determine the experienced donors with a strong willingness to donate blood by a ranking score based on personalized donation data and demographical details, significantly improve the recruitment rate of blood donors and help blood agencies to maintain the blood supply in emergencies.

## Introduction

It is evident that China has made significant advancements in increasing blood donation. From 1998 to 2020, the number of voluntary blood donation and blood collection volume has witnessed growth for 22 consecutive years, which effectively ensured the safety, quality, and availability of blood and improved the public’s confidence in social welfare. Although the amount of donated blood increased continuously at present, the blood supply in China was still in a state of "tight balance"^1^, the security for blood in clinical use was often under pressure for specific seasons, regions, and blood groups, especially in some special scenes, such as in natural disasters, human-induced disasters, public health events, social security incidences, and major events^2–4^, the blood supply was facing great challenges. Therefore, how to take effective measures to recruit more blood donors and prevent blood shortage was the key to dealing with these challenges which called for innovation in the traditional recruitment and promotional approach.

Machine learning is the science of getting computers to act without being explicitly programmed. Cutting-edge ML models can fit high-order relationships between covariates and outcomes in a vast amount of data, which play an important role in a wide variety of complex medical problems and usually perform better than traditional statistical analysis especially when analyzing big medical data^5–7^. Besides, considering that blood data has a large number of samples and full features, ML models can approximate non-linear relationships infinitely, exploring hidden patterns between donors’ intentions and donors’ features. That’s why we chose ML models rather than traditional models. Today, the application of ML technology provides us a way to solve transfusion problems. Several approaches to ML are used in the field of blood transfusion, e.g. the identification of biomarkers of RBC storage quality^8–10^, prediction of RBC transfusion before the surgery^11,12^, and post-transfusion efficacy^13^. However, no studies have been conducted to recruit blood donors using a ML model. The purpose of this study was to explore the factors affecting blood donation behavior and the determinants of donors’ intentions to donate blood with the use of the blood donation big data and machine learning methods, and then develop novel recruitment models to rank donor’s donation will and provide important insights into donors’ decision-making, which could be used by blood agencies to enable personalized precision recruitment, prevent blood shortage, and benefit transfusion patients.

## Methods

### The setting, data sources, and study sample

This study was approved by the ethics committee and conducted at Yangzhou Blood Station from February 1, 2019, to September 30, 2021. The research subject of this paper was donors who spontaneously (non-group) donated whole blood in Yangzhou, China. The dataset was collected from 95,476 donors who received the recruitment SMS from April 8, 2016, to July 3, 2019, excluding the data of platelet donors, donors organized by group, and donors whose key variables were null and abnormal values. All data were de-identified by the removal of 6 identifiers, including name, ID number, affiliation, phone number, E-mail, and family address. When some attribute values were missing, the default values were used for supplement.

### Development of a characteristic database

Based on the hypothesis that donation data could be used to rank and predict donors’ intentions to donate blood, we established a characteristic database of donors to train, test, and compare multiple ML algorithms, by collecting the donation information of each donor over the years, designing and calculating its characteristics according to its blood donation. This database included 13 features as follows: age, gender, blood group, education, living status, occupation, recent and total donation volume, donation times, interval, reaction, blood qualification, and donation frequency that was calculated by equation (Last blood donation date − first blood donation date)/Blood donation times. We constructed the characteristics of effective blood donors and ineffective blood donors before sending SMS and got the training data of the model. With the help of previous SMS recruitment data, we could track the donors receiving the SMS^14^. If the blood donor has a blood donation record within seven days after the SMS had been sent, it is considered an effective blood donor, otherwise, it is an invalid blood donor like the SMS recipient without blood donation.

### Machine learning algorithms

We tuned main hyperparameters of regression, random forest, SVM, DNN, and XGBoost in two steps: (1) we set a wide range of parameters and search for some possible better options; (2) we compared each option and got the best key hyperparameters. The models and their best hyperparameters were as follows.

Decision tree^15^ and linear regression model^16^ were basic models, applied in various tasks, they could be the foundation of other models. Specifically, we constructed a tree with each node representing different features, then it could decide its division standard by calculating its division benefit. Then we tuned the hyperparameters to get the best performance: criterion = 'gini', max_features = None, max_depth = None. Just the same as the decision tree, the linear regression model calculated the error and then update its parameters, and its key hyperparameters were tuned to the best: penalty = ‘l1’, solver = ‘liblinear’.

XGBoost^17^ and RF^18^ were typical ensemble models based on boosting and bagging, respectively. By assembling various basic trees, they tended to have a better performance. To be more specific, the bagging method trained its basic trees with a randomly sampled blood donors dataset, simultaneously, while boosting method trained its basic trees with the whole dataset, but in a serial. XGBoost’s tuned key hyperparameters are max_depth = 50, min_child_weight = 1.5, subsample = 1; RF’s were max_depth = None, min_samples_split = 2, min_samples_leaf = 1.

SVM^19^ was a classic binary classification model, it could divide samples into two classes by maximizing their soft margin. Firstly, we had to design our optimization goal, which was to maximize the soft margin, which could divide samples into two classes. Then we updated the parameters of this margin by calculating the nearest vectors to the margin, which were so-called support vectors. We tuned SVM’s key hyperparameters and get the best parameters as follows: C = 1, gamma = ‘scale’.

KNN^20^ was also a classification model, but not limited to binary classification. It divided samples into different classes by calculating their surrounding samples with clear labels. We should know that this was not a neural network model. It adopted a naive thought: you tended to be like friends and family surrounding you. It’s best key hyperparameters were n_neighbors = 5, weights = ‘uniform', algorithm = 'auto'.

As to DNN^21^, it was a powerful modern model based on the neural network. We had to design its loss function first, which calculated the loss between prediction and ground truth. Then the network could backpropagate this loss and update the parameters of whole neural networks. We found its best key hyperparameters are alpha = 0.0001, activation = ‘relu’, and solver = ‘adam’.

### Model development and validation

We first developed seven ML algorithms, including XGBoost, RF, DNN, SVM, KNN, decision tree, and linear regression models that were all constructed and initialized according to the official publication of sklearn^22^: all the experimental settings followed classic publications and the training processes were typical sklearn training processes. Then, seven ML models were compared in the training-test dataset in which 95,476 samples were randomly split at 70% and 30% of donation records for the training and test dataset respectively. The most important features were screened out and ranked by their importance in the trained model to classify an effective blood donor. Lastly, the accuracy of three better models was validated by the tenfold cross-validation method according to blood groups for evaluating whether the model had good generalization ability. As the characteristics of donors were input into the models, which could give the ranking score in the expected output to recognize and select effective donors, once the model training was completed.

### Recruitment of experienced blood donors

The donors whose features were consistent with donor eligibility requirements were queried in the BIMS and the characteristics of each donor were obtained from the characteristics database of donors. The basic attributes of each donor were inputted into the trained model to obtain the expected output of effective donors, which were ranked and determined according to the output results. Then, the proposed models were applied alone or in combination to prospective validations: the recruitment SMS were sent to the predictive blood donors recommended with higher ranking and their donation records were collected within seven days after the SMS had been sent, routine method was set as control.

### Data processing and statistics

All the statistical analysis was completed using IBM SPSS version 20.0 software. The categorical comparisons between groups were made by the Chi-square or Fisher’s exact test. The Kruskal–Wallis test was applied in the case of non-normally distributed data, P values of less than 0.05 were considered significant.

### Ethics approval

The Ethics Committee of Jiangsu Province Blood Center approved the study protocol on the basis that our study did not involve the privacy of blood donors, and all methods in modelling and in recruitment were performed in accordance with the relevant guidelines and regulations, and that all donors had previously signed a consent form.

## Results

### Overview of the experimental design

**Figure 1:**
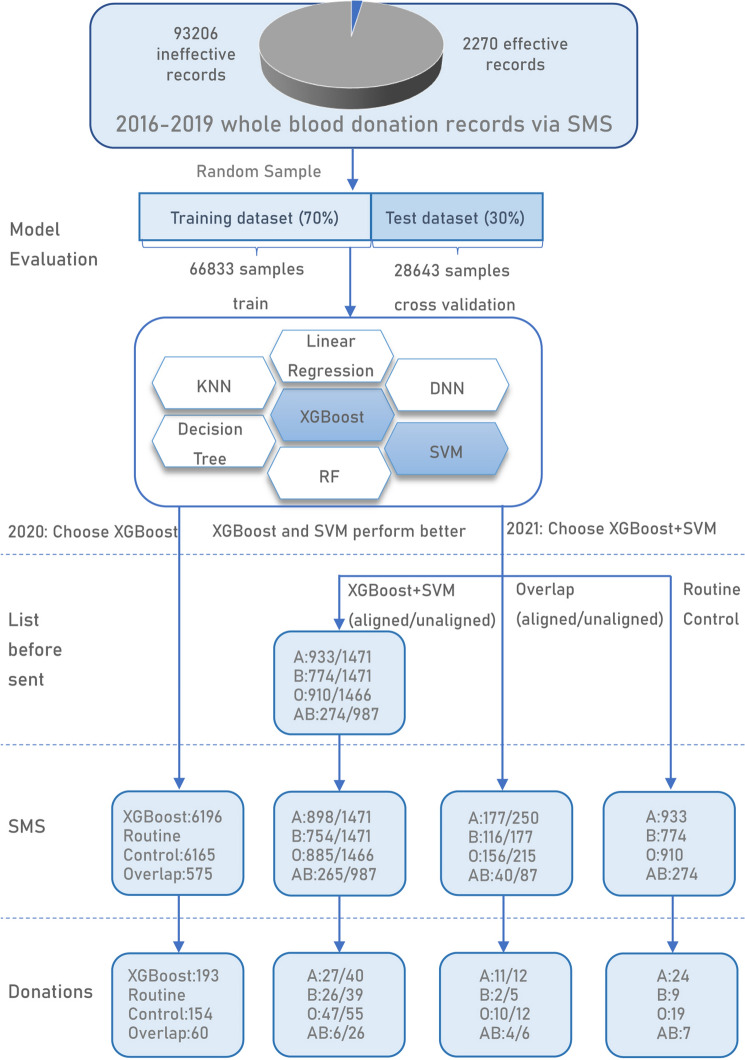
Workflow of the ML algorithm design and blood donor recruitment flowchart. The overall workflow consisted of two main parts: (1) Model construction and inspection. Data were collected from 95,476 donation records via SMS recruitment and were used to develop seven ML models, which were trained and evaluated using random selection with certain ratio and tenfold cross-validation method. (2) Machine learning-based recruitment of blood donors via SMS. Two prospective studies of blood donor recruitment were conducted using machine learning models compared with parallel control of conventional methods as well as self-control of subgroups. In 2020, the donors of four blood groups were recruited using the XGBoost model, compared with routine method as control; in 2021, donors of blood group A, B, O and AB were recruited solely with the combined XGBoost and SVM model. In addition of the comparation with the routine method, the top-ranked group, where the same number of recruitment SMS successfully sent occurred as in the conventional model, was studied against the bottom-ranked group in the machine learning model.

The schematic illustration of our experimental design was shown in Fig. 1. In this study, we used the data of the SMS recruitment records in the Yangzhou blood information management system (BIMS) database. The dataset contained 95,476 (37,246 [39.0%] women and 58,230 [61.0%] men; mean [SD] age, 38.9 [10.8] years) SMS recruitment from 2016 to 2019, including 2270 (885 [39.0%] women and 1385 [61.0%] men; mean [SD] age, 41.8 [9.0], 43.3 [9.2] years) effective donor data and 93,206 (36,361 [39.0%] women and 56,845 [61.0%] men; mean [SD] age, 38.1 [10.5], 39.3 [10.9] years) ineffective donor data. These data were collected to develop seven ML recruitment models which were tested and compared via the training-test dataset and standard tenfold cross-validation experiments. Then, we conducted prospective studies of blood donor recruitment during the COVID-19 pandemics in 2020 and 2021 using two models that performed better in the evaluations (Fig. 1).

### ML modelling

**Figure 2:**
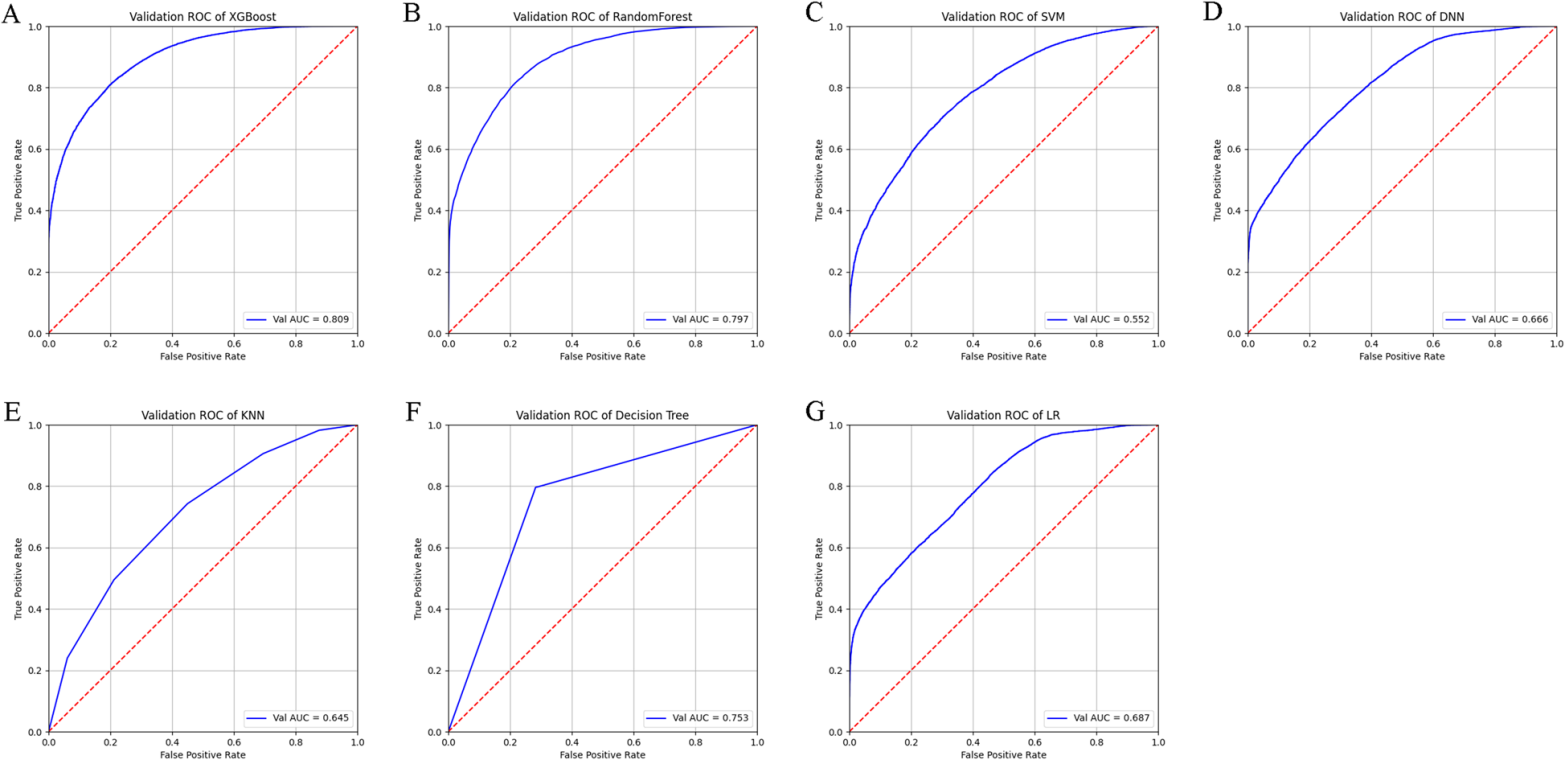
Comparisons of the performance of the seven ML models. The seven ML models were compared against each other based on their ROC curves, and their performances were evaluated by several important metrics such as AUC, accuracy, precision, recall, and f1 score, whose values of Mean and 95% CI were shown in Table S1. (**A**) ROC curves of the XGBoost model, AUC [mean]: 0.809 (0.806–0.811). (**B**) ROC curves of the RF model, AUC [Mean]: 0.797 (0.795–0.800). (**C**) ROC curves of the SVM model, AUC [mean]: 0.552 (0.547–0.557). (**D**) ROC curves of the DNN model, AUC [mean]: 0.666 (0.607–0.724). (**E**) ROC curves of the KNN model, AUC [mean]: 0.645 (0.640–0.650). (**F**) ROC curves of the Decision Tree model, AUC [mean]: 0.753 (0.748–0.758). (**G**) ROC curves of the LA model, AUC [mean]: 0.687 (0.684–0.690).

**Figure 3:**
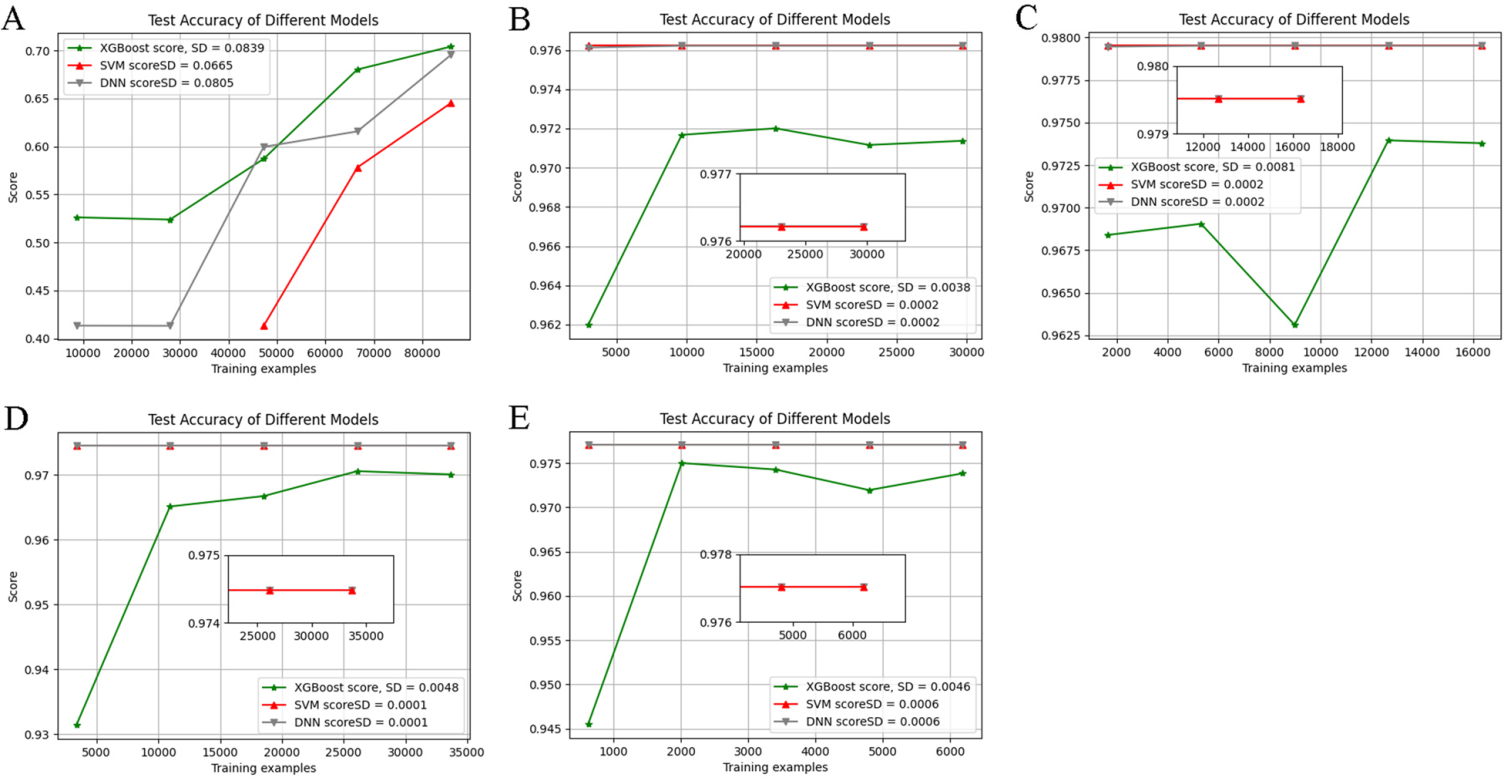
Test accuracy of different models for blood donor recruitment. The three top/median performing algorithms (XGBoost, SVM, and DNN) validated in the training-test dataset were trained and evaluated by tenfold cross-validation method in different datasets. The highest accuracy scores were achieved by XGBoost in the full ABO dataset, and by SVM in the four individual blood group datasets. (**A**) The accuracy was calculated by XGBoost, SVM, and DNN algorithms respectively in the full ABO dataset. (**B**) The accuracy was calculated by XGBoost, SVM, and DNN algorithms respectively in the A blood group dataset. (**C**) The accuracy was calculated by XGBoost, SVM, and DNN algorithms respectively in the B blood group dataset. (**D**) The accuracy was calculated by XGBoost, SVM, and DNN algorithms respectively in the O blood group dataset. (**E**) The accuracy was calculated by XGBoost, SVM, and DNN algorithms respectively in the AB blood group dataset.

Seven ML recruitment models were developed, including XGBoost, RF, DNN, SVM, KNN, decision tree, and linear regression models (Fig. 2). Compared with the other ML algorithms, XGBoost and SVM models achieved the best performance in the training-test dataset, with the highest AUC (0.809), accuracy (0.815), precision (0.840), and F1 (0.843) of XGBOOST and recall (0.991) of SVM, but these of DNN were in the middle (Table S1). However, when validating the accuracy of the three better algorithms with the tenfold cross-validation method, we found that SVM and DNN algorithms outperformed XGBoost in individual blood group validation, except for the validation in the total blood group dataset (Fig. 3).

### Feature importance ranking in XGBoost prediction of donors’ intentions to donate blood

**Figure 4:**
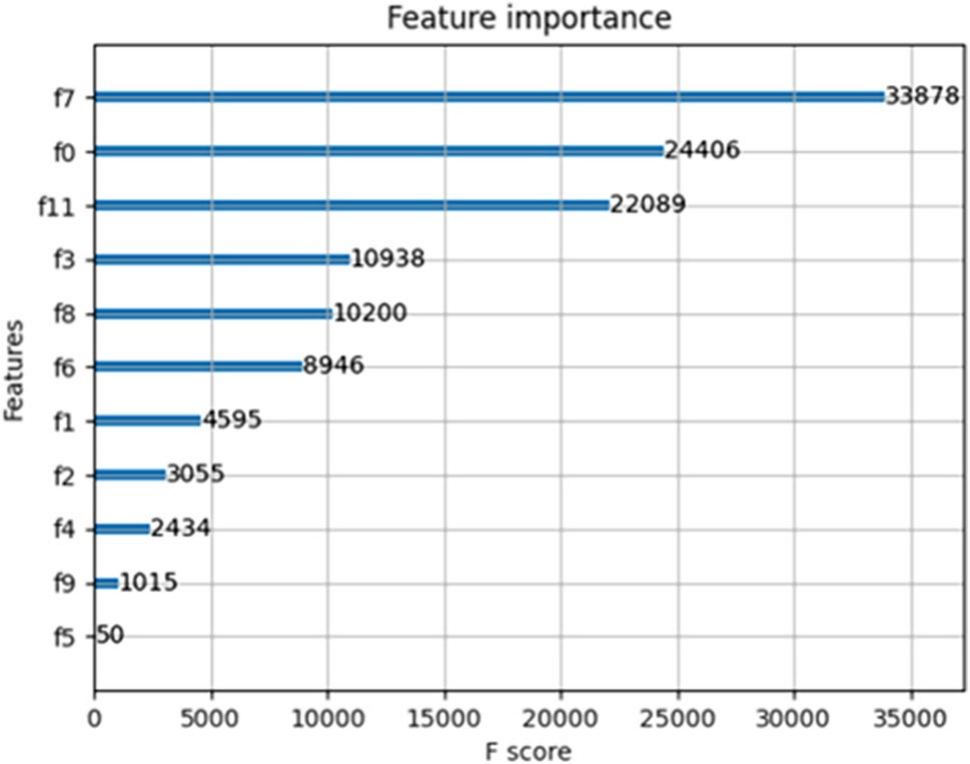
Feature importance of donors for recruitment models. The trained XGBoost model automatically calculated feature importance with a f(eature) score on 13 features associated with willingness to donate blood, we could see that the plot shown F7 (donation interval) had the highest importance and F5 (blood test) had the lowest importance. The features such as donation interval, age, and donation frequency having a strong correlation with blood donors’ intention to donate blood, were considered more important than other features. Besides, total donation volume, education level and professional occupation also played important roles in our recruitment model. f7: Donation interval, f0: Age, f11: Donation frequency, f3: Total donation volume, f8: Education level, f6: Professional occupation, f1: Gender, f2: Recent blood donation, f4: Blood donation times, f9: Living status, f5: Blood test.

A total of 13 features were used to construct the prediction models, including age, gender, blood type, recent donation volume, total donation volume, donation times, donation interval, donation frequency, blood qualification, education, living status, occupation, and blood donation reaction. The value of each feature’s importance was calculated by summing the division times of the feature in each decision tree^23^, and the most important features were donation interval, age, and donation frequency, followed by total donation volume, education level, professional occupation, finally gender, recent blood donation, blood donation times, living status, and whether the blood test was qualified (Fig. 4) for parameters of different models.

### Donor’s SMS recruitment

Table S2 and Table S3 showed the SMS sending and donation statistics during two outbreaks of COVID-19 in Yangzhou in 2020 and 2021. On 3 February 2020, recruitment messages were successfully sent to 6196 XGBoost model-referred donors, of which 193 people donated blood within 1 week of the message being sent, compared to 6165 successful recruitment messages sent using the routine method, with 154 people donating blood within 1 week. The number of overlapping SMS and blood donors was 575 and 60 respectively, and a total of 597 people donated blood in 1 week. In September 2021, 5395, and 2891 recruitment messages were successfully sent by the XGBoost/SVM models and routine method, of which 2802, 489, and 729 were model aligned, overlap aligned, and overlap unaligned respectively, and the corresponding donors were 106/160 (ML aligned/unaligned), 59/59 (RT) and 27/35 (overlap aligned/unaligned) respectively. As 1186 people donated blood in 1 week, the successful rate of SMS recruitment (within 1 week), which was calculated by the equation (donation mobilized via SMS/SMS sent) was 3.1% (193/6 196), 3.8% (106/2 802), and 2.1% ((193–106)/(5395–2802)) respectively in 2020 and 2021 (aligned and unaligned-aligned), while those of RT were 2.5% (154/6165) and 2.0% (59/2891). The blood donation rates (donation mobilized via SMS/total donation) of model, routine method during SMS recruitment week were 32.33% (193/597), 25.80% (154/597) respectively in 2020 and 13.49% (160/1 186), 8.94% (160/1186*106/160), 4.94% (59/1186) (unaligned-aligned) respectively in 2021. In the 2021 data on recruitment by blood group, the ML recruitment rate of each blood group was significantly higher than that by the RT method, both in the aligned and unaligned groups. However, in the recruitment data of the ML unaligned and aligned group, recruitment rates were significantly higher for the four blood groups group as well as for B and O types before alignment group than those of after alignment group, while recruitment rates for A and AB types before and after alignment group were not statistically significant.

### The profile of donors in two SMS recruitments

**Figure 5:**
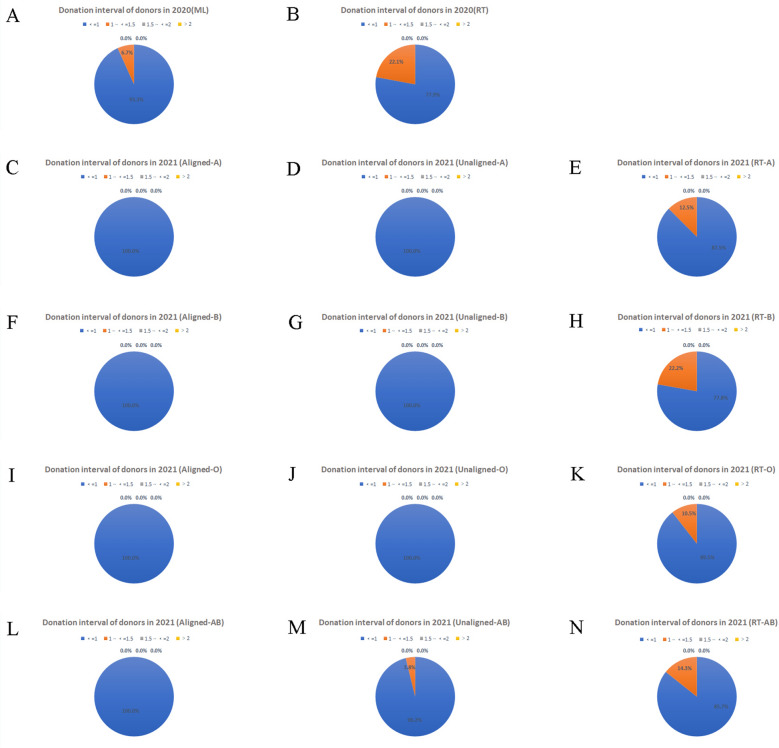
Statistics of donation interval (year) in pandemic recruitment. The interval characteristics of blood donors recruited were analyzed, donors with short intervals made up the majority of donors successfully recruited during the pandemics. (**A**) Donation interval of donors in 2020 (ML). (**B**) Donation interval of donors in 2020 (RT). (**C**) Donation interval of donors in 2021 (Aligned-A). (**D**) Donation interval of donors in 2021 (Unaligned-A). (**E**) Donation interval of donors in 2021 (RT-A). (**F**) Donation interval of donors in 2021 (Aligned-B). (**G**) Donation interval of donors in 2021 (Unaligned-B). (**H**) Donation interval of donors in 2021 (RT-B). (**I**) Donation interval of donors in 2021 (Aligned-O). (**J**) Donation interval of donors in 2021 (Unaligned-O). (**K**) Donation interval of donors in 2021 (RT-O). (**L**) Donation interval of donors in 2021 (Aligned-AB). (**M**) Donation interval of donors in 2021 (Unaligned-AB). (**N**) Donation interval of donors in 2021 (RT-AB).

**Figure 6:**
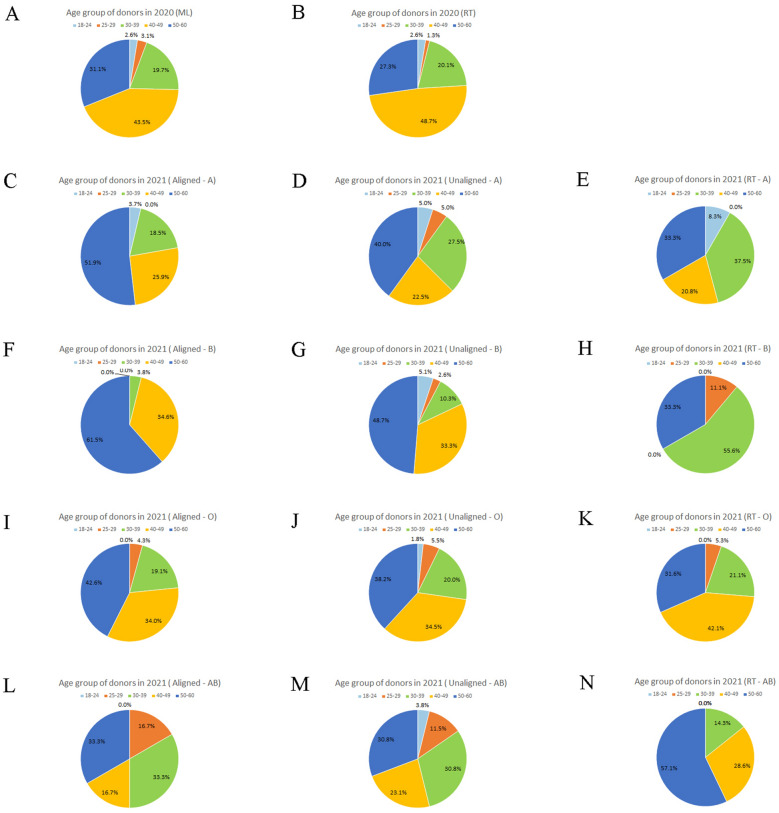
Statistics of donor’s age (years) in pandemic recruitment. The age characteristics of blood donors recruited were analyzed, donors with more than 40 years old made up the majority of donors successfully recruited during the pandemics. (**A**) Ages of donors in 2020 (ML). (**B**) Ages of donors in 2020 (RT). (**C**) Ages of donors in 2021 (Aligned-A). (**D**) Ages of donors in 2021 (Unaligned-A). (**E**) Ages of donors in 2021 (RT-A). (**F**) Ages of donors in 2021 (Aligned-B). (**G**) Ages of donors in 2021 (Unaligned-B). (**H**) Ages of donors in 2021 (RT-B). (**I**) Ages of donors in 2021 (Aligned-O). (**J**) Ages of donors in 2021 (Unaligned-O). (**K**) Ages of donors in 2021 (RT-O). (**L**) Ages of donors in 2021 (Aligned-AB). (**M**) Ages of donors in 2021 (Unaligned-AB). (**N**) Ages of donors in 2021 (RT-AB).

**Figure 7:**
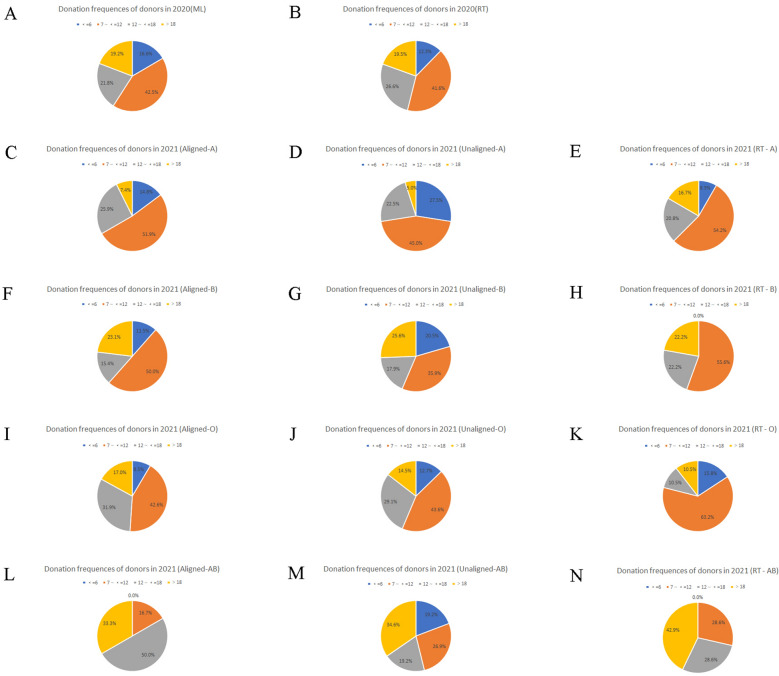
Statistics of donation frequency (months/times) in pandemic recruitment. The donation frequency characteristics of blood donors recruited were analyzed, donors with donation frequency between 7 and 12 months/times made up the majority of donors who were successfully recruited during the pandemics. (**A**) Donation frequency of donors in 2020 (ML). (**B**) Donation frequency of donors in 2020 (RT). (**C**) Donation frequency of donors in 2021 (Aligned-A). (**D**) Donation frequency of donors in 2021 (Unaligned-A). (**E**) Donation frequency of donors in 2021 (RT-A). (**F**) Donation frequency of donors in 2021 (Aligned-B). (**G**) Donation frequency of donors in 2021 (Unaligned-B). (**H**) Donation frequency of donors in 2021 (RT-B). (**I**) Donation frequency of donors in 2021 (Aligned-O). (**J**) Donation frequency of donors in 2021 (Unaligned-O). (**K**) Donation frequency of donors in 2021 (RT-O). (**L**) Donation frequency of donors in 2021 (Aligned-AB). (**M**) Donation frequency of donors in 2021 (Unaligned-AB). (**N**) Donation frequency of donors in 2021 (RT-AB).

Figures 5, 6, 7 revealed the difference in the most important features of blood donors recruited by the ML model and the conventional method in 2020 and 2021. In both 2020 and 2021, neither the ML model nor the conventional method recommended blood donors with intervals longer than 1.5 years, but in 2020 the proportion of ML model recommended donors with intervals within 1 year was 93.3%, compared to 77.9% for the conventional method. In the 2021 recruitment, in addition to 3.8% of AB blood group donors with an interval of 1–1.5 years, the interval of the other three blood groups donors recommended by the ML model was less than 1 year, while the proportion of A, B, O, and AB blood group donors with an interval of 1–1.5 years were 12.5%, 22.2%, 10.5%, and 14.3% respectively in the conventional method (Fig. 5). In the two recruitments of 2020 and 2021, the majority of blood donors recommended by machine learning and conventional methods were over 40 years old, except for the conventional control group for blood group B. And the top-ranked donors in terms of greater willingness to donate (the alignment group) had an even higher proportion of donors over 50 years old (Fig. 6). The data on donor frequency in the 2 recruitments showed that, except AB blood groups, machine learning and conventional methods recommended mostly donors who donated no more than 12 months/time, while among the top-ranked donors with greater intention to donate blood (the alignment group), those who donated 7–12 months/time were predominant (Fig. 7).

The profile of donors with greater willingness to donate in pandemic recruitments was those who were predominantly characterized by men, of Han ethnicity, with a blood donation interval of less than 1 year, older than 40 years old; they usually donated blood once or twice a year with a total donation volume over of 1000 ml, and less than high school or university-level education, whose occupations were employees, workers, and medical staff. Last but not least, they were registered residents and regular donors whose donation times were between 4–20 times, who chose to donate 400 ml of blood, and who had no adverse reactions during the past donation with the qualified rate of the blood test greater than 95% (Table S4).

## Discussion

The city of Yangzhou, located in the eastern part of China, with a population of 4.3 million and a perfect BIMS which gathered about 800,000 local blood donation records, has experienced 2 lockdowns in the last 2 years due to the unprecedented coronavirus pneumonia outbreaks. With the closure of Wuhan on 23 January 2020 and the rapid evolution of COVID-19 from Wuhan, Yangzhou initiated the response mechanism with multisectoral involvement in joint prevention and control measures as in all Chinese cities. The closed-off management hurt blood donation and local inventory less than 2 weeks after the Chinese New Year holiday. In addition, Yangzhou had been sealed off since 31 July 2021 because of the new wave of the coronavirus pandemic, the neighborhood lock-down policy was implemented in both city and rural areas of Yangzhou for epidemic prevention and control, and 7 rounds of nucleic acid testing for all residents plus 20 rounds of testing for key populations with over 30 million tests were launched before 9 September when the neighborhood lock-down policy was initially lifted. These crises caused an unprecedented impact on blood transfusion and collection with large-scale disruption to both the supply and demand for blood.

Although the success rate of SMS recruitment was low, the recruitment information could be sent to a large number of previous donors in a very short time and a great coverage area, making it easier for blood stations to carry out emergency recruitment. In response to the blood shortage, we used some novel recruitment models based on artificial intelligence in SMS recruitment during two pandemics by integrating 95,476 SMS recruitment data and donation records registered during the last 4 years (2016–2019), and analyzed the records of each subjected donor. Considering the complexity of features, hidden nonlinear and feature patterns of large-scale blood donation data, modern ML models^21^ were the most suitable solutions for providing a ranked recommended donor list, e.g. XGBoost^24^ and SVM^25^, as two classic models for data mining and machine learning, which had strong learning ability and extensibility. In general, for a large number of historical blood donation data of 100,000 levels, the XGBoost model could learn the hidden patterns and fit the characteristics of blood donors well, scoring every sample with certain features, which was more suitable for making recommendations^26^ according to the score. To be more specific, every potential donor could get a floating-point number between 0 and 1, indicating how much the donor would like to donate blood and the donor could be ranked with the personnel score meaning the probability^27^ that the potential donor’s intention to donate blood, the bigger the number, the more the donor wanted to donate blood. Moreover, XGBoost and SVM were based on a variety of mature frameworks^28^, which could make full use of the superiority of model structure and modern high-performance hardware for fast calculation and recommendation, reducing the time consumption of model training and prediction, and also by adjusting hyperparameters^29^ and adding regular terms^30^ to make them suitable for different tasks in various fields. As for the impact of performing ‘subsample = 1.0’ in the XGBoost method, we have following opinions and evidences. By applying subsample = 1.0, each basic tree in XGBoost will be trained with the whole training set in serial. If the subsample < 1.0, the trees may not learn enough knowledge. Noteworthy, we do not need to set subsample < 1 since our dataset contains sufficient samples for donors intentions prediction task to avoid overfitting. An important conclusion in statistical learning about overfitting is: the upper bound of the discrepancy between approximation error and estimation error decreases with the increase of sample size^31^. We collected more than 60,000 samples in the training set. This set contains abundant information and features, thus significantly improving the generalization ability of XGBoost and help prevent overfitting. To prove that our training set contains enough samples to avoid overfitting, we conduct an experiment where performance of XGBoost in the testing set changes with sample sizes and subsample of the training set. The dataset used here is the same as the dataset we mentioned above which contains 95,476 samples. We have randomly sampled 1000 to 50,000 training samples and set subsample range from 0.1 to 1.0. Then we train XGBoost and test it on a fixed testing set with more than 20,000 samples. When training sample sizes is small, such as 1000, XGBoost achieves best performance when subsample = 0.8. However, when training sample sizes increase to 5000 or more, the performance of XGBoost in the testing set improves significantly with the increasing value of the subsample, XGBoost performs best only when subsample = 1.0. In conclusion, when we have enough training samples here (above 5000), overfitting is no longer a concern, therefore we can safely set subsample to 1. Finally, we developed our recruitment model which could evaluate each potential donor’s donation will and level of his willingness.

In the process of selecting the modelling subjects, we first excluded donors giving simultaneously both platelets and whole blood because the donation cycle and duration were different. Then, blood donors organized by the group were excluded as their inclusion would weaken the individual recruitment principle. In our modelling process, we found that XGBoost had the best parameters in the training-test dataset, but its accuracy was inferior to those of the SVM and DNN models in the 10-fold cross-validation of the single blood group. These results suggested different strategies for using different models in particular recruitment. Therefore, we constructed integrated modelling of the XGBoost and SVM models in the recruitment of the 4 individual blood groups in 2021, they generated respectively a ranked recommendation list with the recommended values of each donor, and those with the highest recommended values were selected to be the final recruitment targets for generating the recommendation list. It should be stressed that the number of sent messages is largely reduced thanks to our prevention and precision strategy, which helped blood agencies make personalized recruitment plan, improve recruitment efficiency, and save time and money.

During the pandemic in 2020, some blood agencies in China carried out emergency recruitment by SMS: The Guangzhou Blood Center sent 432,396 recruitment SMS to experienced blood donors on January 30th^32^; From January 21 to March 24, 2020, more than 670,000 recruitment-related short messages were distributed by the Sichuan Provincial Health Commission^33^; On January 31th and February 26th, a total of 49,140 recruitment messages were issued respectively by the Dalian Blood Center^34^; From January 23th to February 8th, 38 blood agencies sent 163,791 recruitment texts to donors of Zhejiang Province^35^. The SMS success rate of Guangzhou, Dalian, and Zhejiang were 0.66–1.14% (within 1 week), 2.2% (no time specified), and 3.2% (no time specified) respectively. We used the model developed for SMS recruitment in 2 coronavirus pandemics of 2020 and 2021 respectively. Given that the 2020 outbreak was in the middle of the Chinese New Year and the winter break for students and those students were locked out of school in the 2021 recruitment period, we excluded students from the list of 2 blood donor recruitments. Meanwhile, the 2 ML recruitments were compared with the Yangzhou routine method as control, whose recruitment lists were those who had donated blood from 6 months ago. For the first recruitment in 2020, the XGBoost model was used to recruit donors of all ABO blood groups, while the XGBoost combined with the SVM model was implemented in 2021 to recruit donors of the ABO blood group alone. Although the SMS recruitment rate during the pandemic reported by the literature could not be compared reciprocally under the same conditions, our SMS recruitment rate during the epidemic was significantly higher than the average recruitment rate of 2.4% (2270/95,476) during 2016–2019 in Yangzhou BIMS, it is suggested that the blood donation willingness of blood donors during the pandemic is higher than that of donors in routine SMS recruitment. Seeing that the donor's number in the list aligned with the ML model was 1.80 (106/59) times higher than that of the routine method and 1.96 (106/(160–106)) times higher than that of the remaining part, we believed that it was feasible to use the recommended value generated by AI to predict the level of blood donation intention. Due to the exclusion of students from the outbreak recruitment list, it was not yet possible to conclude that older blood donors were the main target for emergency recruitment, but the interval and frequency of donations were the primary consideration for recruitment.

This study had several limitations. First, this was a single-center, observational study, with the inherent limitations that come from assessing only by one blood agency; Second, the students were excluded from the list of 2 blood donor recruitments, thus affecting the profile of donors in other emergency recruitments. Third, in ML recruitment by each blood group, the recruitment rates for A and AB types before and after the alignment group were not statistically significant, suggesting that there was much still to improve for ML models. Fourth, a small part of blood donors with missing critical data was excluded from modelling processes, causing selection bias that might exist without considering unknown confounding factors. Lastly, although the ranking scores were used to recognize and select effective blood donors, our models could only recruit experienced blood donors, and could not recruit potential blood donors without blood donation records. As for future work, in addition to performing multi-centers validation, we planned to develop an ML automatic scoring system based on our data of emergency calls through phone and SMS, and active guidance from social media, to provide blood agency a more usable and easy-to-understand recruitment tool.

## Conclusions

Overall, our research provided a positive answer to the question of whether the donors’ intention to donate blood could be predicted from their donation records and developed 7 SMS recruitment models based on machine learning algorithms, which could predict whether an experienced donor was willing to donate blood, and the level of his willingness by a ranking score based on personalized donation data and demographical details. To our knowledge, this was the first prospective study on blood donor recruitment using a comprehensive scoring method based on machine learning, which predicted and ranked the personalized motivation of blood donors. The results of this study contributed to improving our understanding of what features were most important for donation and helping blood agencies to predict potential blood donors, make personalized recruitment plans to meet the patient’s needs, and improve recruitment efficacy with precision tactics to maintain the blood supply in emergencies.

## Supplementary Information

Supplementary Information 1.

Supplementary Information 2.

Supplementary Tables.

Supplementary Information 3.

Supplementary Information 4.

## Data Availability

The source data that support the findings of this study is available from the corresponding authors upon reasonable request.

## Abbreviations

ML: Machine learning
SMS: Short message service
AUC: Area under the receiver operating characteristic curve
XGBoost: Extreme Gradient Boosting
RF: Random Forest
SVM: Support vector machine
DNN: Deep neural networks
KNN: K-Nearest Neighbors
RBC: Red blood cell
BIMS: Yangzhou blood information management system
SD: Standard deviation
RT: Routine

## Code availability

All codes from this study are available from supplementary material.
