# Supplementary material for: Predicting willingness to donate blood based on machine learning: two blood donor recruitments during COVID-19 outbreaks
Source: Sci Rep. 2022 Nov 10;12:19165. doi: 10.1038/s41598-022-21215-2 (PMC9647248; doi:10.1038/s41598-022-21215-2)
Supplement: Supplementary file 3 — Supplementary Tables. [file 41598_2022_21215_MOESM3_ESM.docx]

**Supplementary Material**

Table S1 Statistical parameters of the SMS recruitment by different models

| Method | AUC ^a^ | Accuracy^b^ | Precision ^c^ | Recall^d^ | F1 score ^e^ |
| --- | --- | --- | --- | --- | --- |
|  | Mean(95%CI) | Mean(95%CI) | Mean(95%CI) | Mean(95%CI) | Mean(95%CI) |
| XGBoost | **0.809(0.806-0.811)** | **0.815(0.812-0.818)** | **0.840(0.835-0.845)** | 0.845(0.839-0.851) | **0.843(0.840-0.845)** |
| RF | 0.797(0.795-0.800) | 0.810(0.807-0.812) | 0.818(0.814-0.823) | 0.868(0.862-0.875) | **0.843(0.840-0.845)** |
| SVM | 0.552(0.547-0.557) | 0.628(0.621-0.635) | 0.613(0.607-0.619) | **0.991(0.988-0.994)** | 0.757(0.753-0.762) |
| DNN | 0.666(0.607-0.724) | 0.683(0.633-0.732) | 0.743(0.583-0.903) | 0.763(0.341-1.184) | 0.726(0.580-0.872) |
| KNN | 0.645(0.640-0.650) | 0.661(0.656-0.667) | 0.701(0.696-0.706) | 0.738(0.730-0.747) | 0.719(0.713-0.726) |
| Decision Tree | 0.753(0.748-0.758) | 0.760(0.756-0.765) | 0.797(0.791-0.803) | 0.793(0.786-0.800) | 0.795(0.791-0.799) |
| Linear Regression | 0.687(0.684-0.690) | 0.706(0.702-0.709) | 0.728(0.725-0.731) | 0.796(0.789-0.803) | 0.760(0.757-0.764) |

Note: All models were modeled and set according to the official default initialization of sklearn. Each model was repeated 10 times independently in the training-test dataset to obtain the mean indicator value and 95% confidence intervals, which were compared using non-parametric Kruskal-Wallis test. The bold value is the highest (best) for each method respectively.

a:AUC: χ2=67.405, P=.000; b:Accuracy: χ2=66.770, P=.000; c:Precision: χ2=66.394, P=.000; d:Recall: χ2=63.015, P=.000; e:F1 score: χ2=64.002, P=.000

Table S2 SMS sending and blood donation statistics in 2 years

| \| Year \| ML  (SMS/Donation) \| RT  (SMS/Donation) \| Overlap (SMS/Donation) \| Total Donation \| \| --- \| --- \| --- \| --- \| --- \| \| 2020^a^ \| 6196/193 \| 6165/154 \| 575/60 \| 597 \| \| 2021（Aligned） \| 2802/106 \| 2891/59 \| 489/27 \| / \| \| 2021（Unaligned） \| 5395/160 \| 2891/59 \| 729/35 \| 1186 \| |
| --- | --- | --- | --- | --- | --- | --- | --- | --- | --- | --- | --- | --- | --- | --- | --- | --- | --- | --- | --- | --- |

Note: ML: Machine learning; RT: Routine;

Column aligned: The number of donors whose ranking scores are ranked from high to low for each blood type in the ML model is exactly the same as the number of donors in the routine method;

Column unaligned: The total number of donors in the ML model.

a：The chi-square test of 2020 donations: χ2=168.356，P=.000

Table S3 SMS sending and blood donation statistics in 2021 of four blood groups

| Blood type | ML(SMS/Donation) ^a^ | | RT(SMS/Donation) | | Overlap (SMS/Donation) | |
| --- | --- | --- | --- | --- | --- | --- |
|  | Column aligned^b^ | Column unaligned | Column aligned | Column unaligned | Column aligned | Column unaligned |
| A | 898/27 | 1471/24 | 933/40 | 933/24 | 177/11 | 250/12 |
| B | 754/26 | 1471/9 | 774/39 | 774/9 | 116/2 | 177/5 |
| O | 885/47 | 1466/19 | 910/55 | 910/19 | 156/10 | 215/12 |
| AB | 265/6 | 987/7 | 274/26 | 274/7 | 40/4 | 87/6 |

a:ML vs RT b:Aligned vs Unaligned-aligned

Aligned：

A: Fisher’s Exact Test: P=0.006 A: χ2=0.043，P=0.836

B: Fisher’s Exact Test: P=0.005 B: χ2=6.606，P=0.012

O: χ2=21.606，P=0.000 O: χ2=14.126，P=0.000

AB: Fisher’s Exact Test: P=0.006 AB: χ2=2.455，P=0.117

Unaligned：

A: χ2=7.369，P=0.025

B: Fisher’s Exact Test: P=0.007

O: χ2=15.771，P=0.000

AB: Fisher’s Exact Test: P=0.009

Table S4 The profile of donors in two SMS recruitments of 2020 and 2021

|  | 2020 Recruitment（ML/RT） | | 2021 Recruitment Column aligned（ML/RT） | | | | | | | 2021 Recruitment Column unaligned（ML/RT） | | | | | | | | | |  |
| --- | --- | --- | --- | --- | --- | --- | --- | --- | --- | --- | --- | --- | --- | --- | --- | --- | --- | --- | --- | --- |
| Blood group | Total | | A | | B | | O | | AB | | A | | B | | O | | | AB | | |
| Characteristic | Number | % | Number | % | Number | % | Number | % | Number | % | Number | % | Number | % | Number | % | Number | | % |  |
| Age group (years) |  |  |  |  |  |  |  |  |  |  |  |  |  |  |  |  |  | |  |  |
| 18-24 | 5(4) | 2.59(2.6) | 1(2) | 3.7(8.33) | 0(0) | 0(0) | 0(0) | 0(0) | 0(0) | 0(0) | 2(2) | 5(8.33) | 2(0) | 5.13(0) | 1(0) | 1.82(0) | 1(0) | | 3.85(0) |  |
| 25-29 | 6(2) | 3.11(1.3) | 0(0) | 0(0) | 0(1) | 0(11.11) | 2(1) | 4.26(5.26) | 1(0) | 16.67(0) | 2(0) | 5(0) | 1(1) | 2.56(11.11) | 3(1) | 5.45(5.26) | 3(0) | | 11.54(0) |  |
| 30-39 | 38(31) | 19.69(20.13) | 5(9) | 18.52(37.5) | 1(5) | 3.85(55.56) | 9(4) | 19.15(21.05) | 2(1) | 33.33(14.29) | 11(9) | 27.5(37.5) | 4(5) | 10.26(55.56) | 11(4) | 20(21.05) | 8(1) | | 30.77(14.29) |  |
| 40-49 | 84(75) | 43.52(48.7) | 7(5) | 25.93(20.83) | 9(0) | 34.62(0) | 16(8) | 34.04(42.11) | 1(2) | 16.67(28.57) | 9(5) | 22.5(20.83) | 13(0) | 33.33(0) | 19(8) | 34.55(42.11) | 6(2) | | 23.08(28.57) |  |
| 50-60 | 60(42) | 31.09(27.27) | 14(8) | 51.85(33.33) | 16(3) | 61.54(33.33) | 20(6) | 42.55(31.58) | 2(4) | 33.33(57.14) | 16(8) | 40(33.33) | 19(3) | 48.72(33.33) | 21(6) | 38.18(31.58) | 8(4) | | 30.77(57.14) |  |
| Sex |  |  |  |  |  |  |  |  |  |  |  |  |  |  |  |  |  | |  |  |
| Female | 73(70) | 37.82(45.45) | 10(10) | 37.04(41.67) | 9(2) | 34.62(22.22) | 14(9) | 29.79(47.37) | 1(3) | 16.67(42.86) | 15(10) | 37.5(41.67) | 16(2) | 41.03(22.22) | 19(9) | 34.55(47.37) | 8(3) | | 30.77(42.86) |  |
| Male | 120(84) | 62.18(54.55) | 17(14) | 62.96(58.33) | 17(7) | 65.38(77.78) | 33(10) | 70.21(52.63) | 5(4) | 83.33(57.14) | 25(14) | 62.5(58.33) | 23(7) | 58.97(77.78) | 36(10) | 65.45(52.63) | 18(4) | | 69.23(57.14) |  |
| Race/ethnicity |  |  |  |  |  |  |  |  |  |  |  |  |  |  |  |  |  | |  |  |
| Han | 193(152) | 100(98.7) | 27(24) | 100(100) | 26(9) | 100(100) | 46(19) | 97.87(100) | 6(7) | 100(100) | 40(24) | 100(100) | 39(9) | 100(100) | 53(19) | 96.36(100) | 26(7) | | 100(100) |  |
| National minority | 0(2) | 0(1.3) | 0(0) | 0(0) | 0(0) | 0(0) | 1(0) | 2.13(0) | 0(0) | 0(0) | 0(0) | 0(0) | 0(0) | 0(0) | 2(0) | 3.64(0) | 0(0) | | 0(0) |  |
| Foreign nationality | 0(0) | 0(0) | 0(0) | 0(0) | 0(0) | 0(0) | 0(0) | 0(0) | 0(0) | 0(0) | 0(0) | 0(0) | 0(0) | 0(0) | 0(0) | 0(0) | 0(0) | | 0(0) |  |
| Other | 0(0) | 0(0) | 0(0) | 0(0) | 0(0) | 0(0) | 0(0) | 0(0) | 0(0) | 0(0) | 0(0) | 0(0) | 0(0) | 0(0) | 0(0) | 0(0) | 0(0) | | 0(0) |  |
| Occupation |  |  |  |  |  |  |  |  |  |  |  |  |  |  |  |  |  | |  |  |
| Student | 0(0) | 0(0) | 0(0) | 0(0) | 0(0) | 0(0) | 0(0) | 0(0) | 0(0) | 0(0) | 0(0) | 0(0) | 0(0) | 0(0) | 0(0) | 0(0) | 0(0) | | 0(0) |  |
| Soldier | 0(0) | 0(0) | 0(0) | 0(0) | 0(0) | 0(0) | 0(0) | 0(0) | 0(0) | 0(0) | 0(0) | 0(0) | 0(0) | 0(0) | 0(0) | 0(0) | 0(0) | | 0(0) |  |
| Public functionary | 4(5) | 2.07(3.25) | 1(0) | 3.7(0) | 1(0) | 3.85(0) | 5(1) | 10.64(5.26) | 0(0) | 0(0) | 1(0) | 2.5(0) | 1(0) | 2.56(0) | 5(1) | 9.09(5.26) | 0(0) | | 0(0) |  |
| Medical staff | 14(19) | 7.25(12.34) | 6(2) | 22.22(8.33) | 5(1) | 19.23(11.11) | 2(1) | 4.26(5.26) | 0(0) | 0(0) | 9(2) | 22.5(8.33) | 8(1) | 20.51(11.11) | 6(1) | 10.91(5.26) | 8(0) | | 30.77(0) |  |
| Industrial | 8(5) | 4.15(3.25) | 6(7) | 22.22(29.17) | 2(3) | 7.69(33.33) | 7(5) | 14.89(26.32) | 1(1) | 16.67(14.29) | 8(7) | 20(29.17) | 4(3) | 10.26(33.33) | 8(5) | 14.55(26.32) | 2(1) | | 7.69(14.29) |  |
| Farmer | 16(13) | 8.29(8.44) | 0(0) | 0(0) | 2(1) | 7.69(11.11) | 0(0) | 0(0) | 0(0) | 0(0) | 0(0) | 0(0) | 3(1) | 7.69(11.11) | 0(0) | 0(0) | 0(0) | | 0(0) |  |
| Clerk | 70(58) | 36.27(37.66) | 4(4) | 14.81(16.67) | 5(0) | 19.23(0) | 18(2) | 38.3(10.53) | 1(2) | 16.67(28.57) | 6(4) | 15(16.67) | 10(0) | 25.64(0) | 19(2) | 34.55(10.53) | 7(2) | | 26.92(28.57) |  |
| Teacher | 8(4) | 4.15(2.6) | 1(1) | 3.7(4.17) | 0(0) | 0(0) | 0(1) | 0(5.26) | 0(0) | 0(0) | 1(1) | 2.5(4.17) | 0(0) | 0(0) | 0(1) | 0(5.26) | 1(0) | | 3.85(0) |  |
| Other | 73(50) | 37.82(32.47) | 9(10) | 33.33(41.67) | 11(4) | 42.31(44.44) | 15(9) | 31.91(47.37) | 4(4) | 66.67(57.14) | 15(10) | 37.5(41.67) | 13(4) | 33.33(44.44) | 17(9) | 30.91(47.37) | 8(4) | | 30.77(57.14) |  |
| Educational attainment |  |  |  |  |  |  |  |  |  |  |  |  |  |  |  |  |  | |  |  |
| Less than high school | 52(47) | 26.94(30.52) | 3(2) | 11.11(8.33) | 7(5) | 26.92(55.56) | 5(3) | 10.64(15.79) | 0(1) | 0(14.29) | 7(2) | 17.5(8.33) | 11(5) | 28.21(55.56) | 5(3) | 9.09(15.79) | 1(1) | | 3.85(14.29) |  |
| High school graduate | 46(43) | 23.83(27.92) | 8(9) | 29.63(37.5) | 7(1) | 26.92(11.11) | 12(6) | 25.53(31.58) | 4(4) | 66.67(57.14) | 11(9) | 27.5(37.5) | 10(1) | 25.64(11.11) | 14(6) | 25.45(31.58) | 6(4) | | 23.08(57.14) |  |
| Some college | 68(45) | 35.23(29.22) | 13(12) | 48.15(50) | 8(2) | 30.77(22.22) | 21(9) | 44.68(47.37) | 2(2) | 33.33(28.57) | 18(12) | 45(50) | 13(2) | 33.33(22.22) | 26(9) | 47.27(47.37) | 15(2) | | 57.69(28.57) |  |
| College graduate | 3(1) | 1.55(0.65) | 0(0) | 0(0) | 0(0) | 0(0) | 0(0) | 0(0) | 0(0) | 0(0) | 0(0) | 0(0) | 0(0) | 0(0) | 0(0) | 0(0) | 1(0) | | 3.85(0) |  |
| Other | 24(18) | 12.44(11.69) | 3(1) | 11.11(4.17) | 4(1) | 15.38(11.11) | 9(1) | 19.15(5.26) | 0(0) | 0(0) | 4(1) | 10(4.17) | 5(1) | 12.82(11.11) | 10(1) | 18.18(5.26) | 3(0) | | 11.54(0) |  |
| Residential status |  |  |  |  |  |  |  |  |  |  |  |  |  |  |  |  |  | |  |  |
| Registered residence | 188(151) | 97.41(98.05) | 27(22) | 100(91.67) | 26(8) | 100(88.89) | 47(18) | 100(94.74) | 6(7) | 100(100) | 40(22) | 100(91.67) | 39(8) | 100(88.89) | 54(18) | 98.18(94.74) | 25(7) | | 96.15(100) |  |
| Non registered residence | 4(2) | 2.07(1.3) | 0(2) | 0(8.33) | 0(1) | 0(11.11) | 0(1) | 0(5.26) | 0(0) | 0(0) | 0(2) | 0(8.33) | 0(1) | 0(11.11) | 1(1) | 1.82(5.26) | 1(0) | | 3.85(0) |  |
| Other | 1(1) | 0.52(0.65) | 0(0) | 0(0) | 0(0) | 0(0) | 0(0) | 0(0) | 0(0) | 0(0) | 0(0) | 0(0) | 0(0) | 0(0) | 0(0) | 0(0) | 0(0) | | 0(0) |  |
| Recent_donation volume(ml） |  |  |  |  |  |  |  |  |  |  |  |  |  |  |  |  |  | |  |  |
| 200 | 0(0) | 0(0) | 0(0) | 0(0) | 0(0) | 0(0) | 0(0) | 0(0) | 0(0) | 0(0) | 0(0) | 0(0) | 0(0) | 0(0) | 0(0) | 0(0) | 0(0) | | 0(0) |  |
| 300 | 49(37) | 25.39(24.03) | 6(4) | 22.22(16.67) | 4(1) | 15.38(11.11) | 13(4) | 27.66(21.05) | 0(1) | 0(14.29) | 8(4) | 20(16.67) | 10(1) | 25.64(11.11) | 17(4) | 30.91(21.05) | 6(1) | | 23.08(14.29) |  |
| 400 | 144(117) | 74.61(75.97) | 21(20) | 77.78(83.33) | 22(8) | 84.62(88.89) | 34(15) | 72.34(78.95) | 6(6) | 100(85.71) | 32(20) | 80(83.33) | 29(8) | 74.36(88.89) | 38(15) | 69.09(78.95) | 20(6) | | 76.92(85.71) |  |
| Total_donation volume(ml） |  |  |  |  |  |  |  |  |  |  |  |  |  |  |  |  |  | |  |  |
| ﹤=1000 | 23(20) | 11.92(12.99) | 1(1) | 3.7(4.17) | 0(0) | 0(0) | 1(2) | 2.13(10.53) | 0(0) | 0(0) | 5(1) | 12.5(4.17) | 4(0) | 10.26(0) | 4(2) | 7.27(10.53) | 6(0) | | 23.08(0) |  |
| 1000～﹤=2000 | 67(46) | 34.72(29.87) | 3(6) | 11.11(25) | 5(4) | 19.23(44.44) | 7(1) | 14.89(5.26) | 0(0) | 0(0) | 8(6) | 20(25) | 10(4) | 25.64(44.44) | 10(1) | 18.18(5.26) | 7(0) | | 26.92(0) |  |
| 2000～﹤=4000 | 51(43) | 26.42(27.92) | 10(11) | 37.04(45.83) | 9(3) | 34.62(33.33) | 22(10) | 46.81(52.63) | 4(4) | 66.67(57.14) | 14(11) | 35(45.83) | 13(3) | 33.33(33.33) | 24(10) | 43.64(52.63) | 10(4) | | 38.46(57.14) |  |
| ＞4000 | 52(45) | 26.94(29.22) | 13(6) | 48.15(25) | 12(2) | 46.15(22.22) | 17(6) | 36.17(31.58) | 2(3) | 33.33(42.86) | 13(6) | 32.5(25) | 12(2) | 30.77(22.22) | 17(6) | 30.91(31.58) | 3(3) | | 11.54(42.86) |  |
| Donation times |  |  |  |  |  |  |  |  |  |  |  |  |  |  |  |  |  | |  |  |
| ﹤=3 | 39(27) | 20.21(17.53) | 2(2) | 7.41(8.33) | 2(2) | 7.69(22.22) | 2(2) | 4.26(10.53) | 0(0) | 0(0) | 8(2) | 20(8.33) | 7(2) | 17.95(22.22) | 6(2) | 10.91(10.53) | 8(0) | | 30.77(0) |  |
| 4～10 | 94(79) | 48.7(51.3) | 11(14) | 40.74(58.33) | 11(5) | 42.31(55.56) | 25(11) | 53.19(57.89) | 4(4) | 66.67(57.14) | 18(14) | 45(58.33) | 19(5) | 48.72(55.56) | 29(11) | 52.73(57.89) | 15(4) | | 57.69(57.14) |  |
| 11～20 | 53(44) | 27.46(28.57) | 14(7) | 51.85(29.17) | 13(2) | 50(22.22) | 17(4) | 36.17(21.05) | 2(3) | 33.33(42.86) | 14(7) | 35(29.17) | 13(2) | 33.33(22.22) | 17(4) | 30.91(21.05) | 3(3) | | 11.54(42.86) |  |
| ＞20 | 7(4) | 3.63(2.6) | 0(1) | 0(4.17) | 0(0) | 0(0) | 3(2) | 6.38(10.53) | 0(0) | 0(0) | 0(1) | 0(4.17) | 0(0) | 0(0) | 3(2) | 5.45(10.53) | 0(0) | | 0(0) |  |
| Donation interval (year) |  |  |  |  |  |  |  |  |  |  |  |  |  |  |  |  |  | |  |  |
| 1st Donation | 0(0) | 0(0) | 0(0) | 0(0) | 0(0) | 0(0) | 0(0) | 0(0) | 0(0) | 0(0) | 0(0) | 0(0) | 0(0) | 0(0) | 0(0) | 0(0) | 0(0) | | 0(0) |  |
| ﹤=1 | 180(120) | 93.26(77.92) | 27(21) | 100(87.5) | 26(7) | 100(77.78) | 47(17) | 100(89.47) | 6(6) | 100(85.71) | 40(21) | 100(87.5) | 39(7) | 100(77.78) | 55(17) | 100(89.47) | 25(6) | | 96.15(85.71) |  |
| 1～﹤=1.5 | 13(34) | 6.74(22.08) | 0(3) | 0(12.5) | 0(2) | 0(22.22) | 0(2) | 0(10.53) | 0(1) | 0(14.29) | 0(3) | 0(12.5) | 0(2) | 0(22.22) | 0(2) | 0(10.53) | 1(1) | | 3.85(14.29) |  |
| 1.5～﹤=2 | 0(0) | 0(0) | 0(0) | 0(0) | 0(0) | 0(0) | 0(0) | 0(0) | 0(0) | 0(0) | 0(0) | 0(0) | 0(0) | 0(0) | 0(0) | 0(0) | 0(0) | | 0(0) |  |
| ＞2 | 0(0) | 0(0) | 0(0) | 0(0) | 0(0) | 0(0) | 0(0) | 0(0) | 0(0) | 0(0) | 0(0) | 0(0) | 0(0) | 0(0) | 0(0) | 0(0) | 0(0) | | 0(0) |  |
| Donation frequency (months/times) |  |  |  |  |  |  |  |  |  |  |  |  |  |  |  |  |  | |  |  |
| ﹤=6 | 32(19) | 16.58(12.34) | 4(2) | 14.81(8.33) | 3(0) | 11.54(0) | 4(3) | 8.51(15.79) | 0(0) | 0(0) | 11(2) | 27.5(8.33) | 8(0) | 20.51(0) | 7(3) | 12.73(15.79) | 5(0) | | 19.23(0) |  |
| 7～﹤=12 | 82(64) | 42.49(41.56) | 14(13) | 51.85(54.17) | 13(5) | 50(55.56) | 20(12) | 42.55(63.16) | 1(2) | 16.67(28.57) | 18(13) | 45(54.17) | 14(5) | 35.9(55.56) | 24(12) | 43.64(63.16) | 7(2) | | 26.92(28.57) |  |
| 12～﹤=18 | 42(41) | 21.76(26.62) | 7(5) | 25.93(20.83) | 4(2) | 15.38(22.22) | 15(2) | 31.91(10.53) | 3(2) | 50(28.57) | 9(5) | 22.5(20.83) | 7(2) | 17.95(22.22) | 16(2) | 29.09(10.53) | 5(2) | | 19.23(28.57) |  |
| ＞18 | 37(30) | 19.17(19.48) | 2(4) | 7.41(16.67) | 6(2) | 23.08(22.22) | 8(2) | 17.02(10.53) | 2(3) | 33.33(42.86) | 2(4) | 5(16.67) | 10(2) | 25.64(22.22) | 8(2) | 14.55(10.53) | 9(3) | | 34.62(42.86) |  |
| Blood test |  |  |  |  |  |  |  |  |  |  |  |  |  |  |  |  |  | |  |  |
| Qualified | 192(152) | 99.48(98.7) | 27(24) | 100(100) | 25(9) | 96.15(100) | 46(19) | 97.87(100) | 6(7) | 100(100) | 40(24) | 100(100) | 38(9) | 97.44(100) | 54(19) | 98.18(100) | 26(7) | | 100(100) |  |
| Non qualified | 1(2) | 0.52(1.3) | 0(0) | 0(0) | 1(0) | 3.85(0) | 1(0) | 2.13(0) | 0(0) | 0(0) | 0(0) | 0(0) | 1(0) | 2.56(0) | 1(0) | 1.82(0) | 0(0) | | 0(0) |  |
| Blood donation reaction |  |  |  |  |  |  |  |  |  |  |  |  |  |  |  |  |  | |  |  |
| Yes | 0(0) | 0(0) | 0(0) | 0(0) | 0(0) | 0(0) | 0(0) | 0(0) | 0(0) | 0(0) | 0(0) | 0(0) | 0(0) | 0(0) | 0(0) | 0(0) | 0(0) | | 0(0) |  |
| No | 193(154) | 100(100) | 27(24) | 100(100) | 26(9) | 100(100) | 47(19) | 100(100) | 6(7) | 100(100) | 40(24) | 100(100) | 39(9) | 100(100) | 55(19) | 100(100) | 26(7) | | 100(100) |  |
